# Advanced passive 3D bioelectronics: powerful tool for the cardiac electrophysiology investigation

**DOI:** 10.1038/s41378-025-00891-w

**Published:** 2025-03-17

**Authors:** Keda Shi, Chengwen He, Hui Pan, Dong Liu, Ji Zhang, Weili Han, Yuting Xiang, Ning Hu

**Affiliations:** 1 Department of Lung Transplantation and General Thoracic Surgery, The First Affiliated Hospital, Zhejiang University School of Medicine, Hangzhou, 310003 China; 2 Department of Chemistry, Zhejiang-Israel Joint Laboratory of Self-Assembling Functional Materials, ZJU-Hangzhou Global Scientific and Technological Innovation Center, School of Medicine, Zhejiang University, Hangzhou, 310058 China; 3 General Surgery Department, Children’s Hospital, Zhejiang University School of Medicine, National Clinical Research Center for Children’s Health, Hangzhou, 310052 China; 4 Department of Obstetrics, the Tenth Affiliated Hospital, Southern Medical University, Dongguan, 523059 China

**Keywords:** Biosensors, Bionanoelectronics

## Abstract

Cardiovascular diseases (CVDs) are the first cause of death globally, posing a significant threat to human health. Cardiac electrophysiology is pivotal for the understanding and management of CVDs, particularly for addressing arrhythmias. A significant proliferation of micro-nano bioelectric devices and systems has occurred in the field of cardiomyocyte electrophysiology. These bioelectronic platforms feature distinctive electrode geometries that improve the fidelity of native electrophysiological signals. Despite the prevalence of planar microelectrode arrays (MEAs) for simultaneous multichannel recording of cellular electrophysiological signals, extracellular recordings often yield suboptimal signal quality. In contrast, three-dimensional (3D) MEAs and advanced penetration strategies allow high-fidelity intracellular signal detection. 3D nanodevices are categorized into the active and the passive. Active devices rely on external power sources to work, while passive devices operate without external power. Passive devices possess simplicity, biocompatibility, stability, and lower power consumption compared to active ones, making them ideal for sensors and implantable applications. This review comprehensively discusses the fabrication, geometric configuration, and penetration strategies of passive 3D micro/nanodevices, emphasizing their application in drug screening and disease modeling. Moreover, we summarize existing challenges and future opportunities to develop passive micro/nanobioelectronic devices from cardiac electrophysiological research to cardiovascular clinical practice.

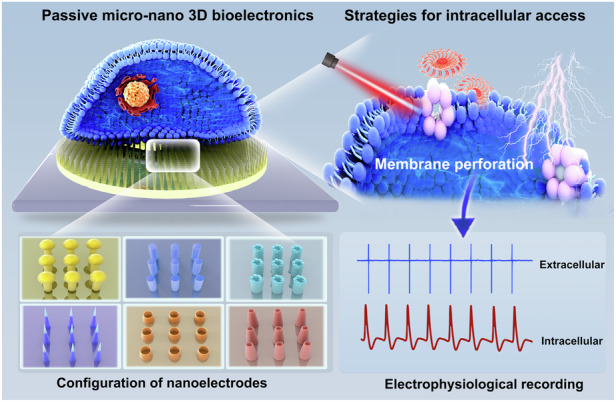

## Introduction

Cardiovascular diseases (CVD) are the leading cause of premature mortality, imposing a substantial burden on population health and socio-economic well-being. The widespread impact of CVD on populations, coupled with the complexity of management, underscores the growing burden on healthcare systems and patients^1–3^. Moreover, drug-induced cardiotoxicity is a significant factor that restricts the development and utilization of new drugs. Many drugs are precluded from clinical application or recalled post-marketing due to adverse cardiac effects. More seriously, drug-induced cardiotoxicity can lead to cardiovascular complications, and in severe cases, may even result in sudden death^4–7^. Less than 30% of the compounds tested in U.S. clinical trials successfully reach commercial availability due to drug-induced cardiotoxicity, which leads to a cycle of wasted time, increased expenses, and resource depletion, highlighting the urgent need for accelerated research into all facets of cardiovascular effects^8^. Electrocardiography, echocardiography, and coronary computed tomography angiography stand as the primary methods to evaluate cardiovascular morphology^9–12^. While providing insights into cardiac function, these diagnostic tools are constrained by the low spatial resolution^13^. To overcome these challenges, cardiomyocyte-based models and sensing technologies are proposed as promising alternatives^14–16^. Establishing a robust cardiomyocyte-based platform is crucial for comprehensive CVD investigation and early anticipation of drug-related cardiotoxicity^17^.

Electrophysiological sensing of cardiomyocytes is a commonly used and important method for characterizing cell status. Electrophysiological methods are widely recognized for their utility in both characterizing and modulating the behaviors of electrogenic cells^18,19^. Upon the occurrence of hereditary or acquired CVDs, the generation and propagation of action potentials (AP) are altered due to changes in the expression, distribution, and characteristics of myocardial ion channels^20^. Thus, evaluating electrophysiological dysfunction at the cell level is crucial to understanding organ dysfunction caused by molecular aberrations^21^. For electrophysiological evaluation, patch clamp is the gold standard for precise, high-fidelity transmembrane potential measurements, offering detailed, high-resolution insights into ion channel properties, which is labor-intensive and low-throughput^22^. Automated systems increase efficiency but often require isolated cells, which can disrupt cardiomyocyte electrical coupling and affect measurement accuracy^23,24^. Extracellular electrodes like planar microelectrode arrays (MEAs) enable noninvasive and easy recording of extracellular APs but cannot capture transmembrane potentials, limiting their ability to reflect detailed arrhythmic features^18,25^. Alternatively, intracellular recordings from three-dimensional (3D) nanoelectrode arrays (NEAs) can accurately measure AP duration, refractory period, and upstroke velocity, providing precise parameters for electrophysiological properties of cardiomyocytes^26–28^. Therefore, NEAs emerge as highly promising tools in the field of cardiovascular research.

Specifically, there exist two major categories of 3D nanodevices: active and passive. As detailed in Table 1, active devices can actively control or modify electrical signals, and offer capabilities such as amplification, signal fidelity, dynamic modulation, and real-time processing, making them suitable for applications that demand high resolution and precision, particularly in electrophysiology and biomedical sensing. However, active NEAs require an external power source to operate. Passive devices, however, are characterized by their simple structure and independence of an external power source, which facilitates easier and more cost-effective design and manufacturing^29^. Furthermore, passive devices often exhibit high biocompatibility, generating minimal heat and causing less impact on biological tissues^30^. Their simplicity also increases stability and durability, with minimal susceptibility to external interference. Additionally, passive devices have low power consumption, making them ideal for miniaturized or implantable applications^31^. Due to the straightforward design and reduced complexity, passive devices are especially suited for large-scale applications requiring mass production^32^. Thus, passive devices are widely used for electrophysiological sensing, particularly where active control is not necessary. Despite the aforementioned advantages, the design and 3D structure of the passive devices are crucial for the quality of intracellular signal acquisition.

**Table 1:** Comparison of passive and active 3D nanoelectrode arrays (NEAs) in cardiac electrophysiology

Feature	Passive 3D NEAs	Active 3D NEAs
Power source	Operation without an external power.	Operation supported by an external power (e.g., amplifiers, signal modulation).
Complexity	Simple design and easy fabrication and integration.	Complex design and fabrication.
Biocompatibility	High biocompatibility without heat generation.	Lower biocompatibility due to active components and heat generation.
Signal amplification	No amplification.	Active signal amplification.
Stability	High stability and durability.	Stability affected by heat generation, power fluctuations, and device complexity.
Scalability	High scalability due to simpler design and fabrication.	Low scalability due to the active component fabrication.
Cost of production	Lower cost due to simpler fabrication processes.	Higher cost due to complex fabrication processes.

As for the fabrication of NEAs, micro-nano-processing and integration with 3D bioelectronics allow the recording of electrophysiological signals from both single cells and their networks^33^. The vertical geometry of NEAs is crucial for strengthening signal detection^34^. The tight envelopment of the cell membrane around vertical electrodes reduces the separation between the cell membrane and electrode, thereby boosting seal resistance^35–37^. Moreover, for bioelectronic devices to achieve a signal-to-noise ratio (SNR) and amplitude comparable to those in patch clamp, establishing direct contact between recording elements and targeted intracellular locations is necessary while minimizing interference with cell membranes^38^. To meet these criteria, it is essential to thoroughly understand the size, shape, mechanical properties, and biochemical interactions at the interface between the cell membrane and the nanodevice^39^. Manufacturing techniques significantly influence nanodevice size, geometry, and the interaction of NEAs with cell membranes. Advances in nanoelectrode technology, particularly through the integration of Complementary Metal Oxide Semiconductor (CMOS) technology, have significantly enhanced the ability to record and modulate cellular electrical activity on a large scale^40^. Furthermore, by enabling parallel recordings from individual cells and networks, these innovations improve the precision and scalability of electrophysiological studies.

In this review, we will delve into the critical role of arrhythmia as an early diagnostic marker for CVDs, emphasizing the importance of intracellular cardiomyocyte electrophysiological analysis for diagnosis. With the advent of new electrophysiological detection techniques, 3D NEAs have gained prominence for both in vivo and in vitro applications. Here, we focus on the potential of passive 3D NEAs to improve early CVD detection through arrhythmia diagnosis. We discuss the essential aspects that impact electrophysiological recordings using passive 3D NEAs: the manufacturing and configuration of passive 3D NEAs, as well as strategies for intracellular access. We then explore the application of passive 3D NEAs in modeling CVD and precision drug screening. Further, we discuss future research directions in the development of passive 3D NEA structures, including advances in CMOS technology, the integration of micro and nanoelectrodes, multiparameter detection, human organoid models, and the convergence of sensing and regulatory functions.

## Manufacturing and configuration of passive 3D NEAs

**Fig. 1:**
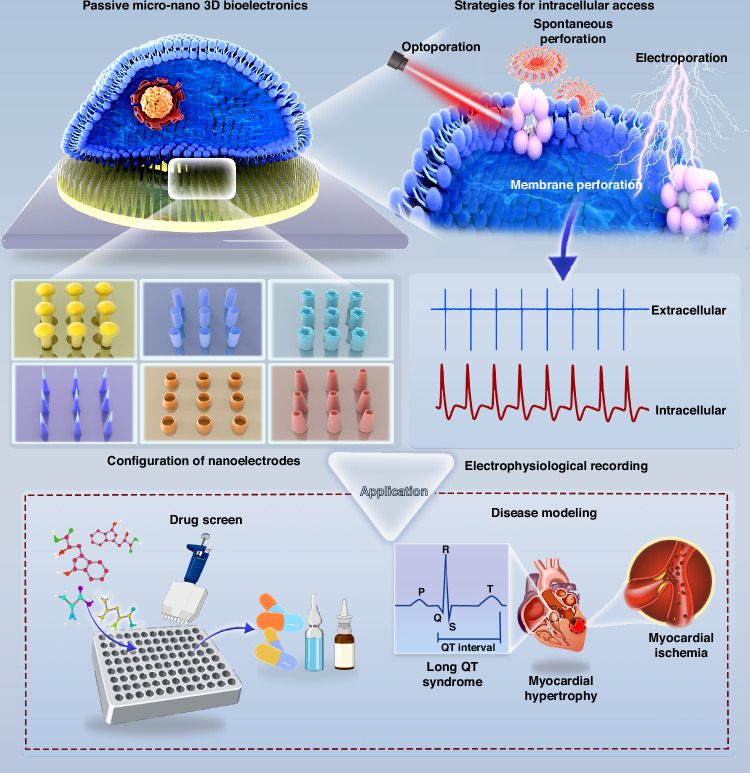
Advanced passive 3D bioelectronics system for studying cardiac electrophysiology. Physical regulation requires that the electrode penetrates the cell membrane through physical techniques, primarily electro- and opto-poration. Chemical regulation entails gaining intracellular access through chemical methods, such as modifying the electrode surface with a phospholipid bilayer

Manufacturing processes are fundamental in designing and fabricating 3D nanostructures for passive 3D micro-nano bioelectronic platforms and advancing intracellular electrophysiological recordings^41^. Recent studies have emphasized that the placement, geometry, and dimensions of nanoelectrodes greatly affect electrophysiological detection, with geometry as key to effective cell-device coupling, particularly in 3D configurations^42–44^. Different 3D geometric shapes have been explored for recording electrophysiological signals in cardiomyocytes, each presenting unique innovations, advantages, and challenges^38^. Here, we provide an in-depth analysis of these strategies to enhance the understanding of nanoelectrode fabrication processes for the precise manufacturing of passive 3D NEAs (Fig. 1).

### Advances in passive 3D nanoelectrode fabrication

**Fig. 2:**
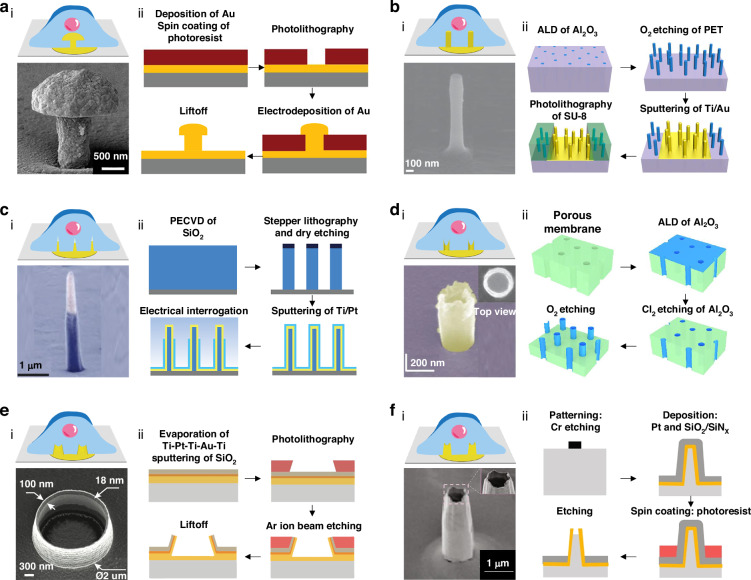
Innovative designs for electrode-tissue interfaces. The fabrication processes and representative configurations of the 3D electrode-tissue interface with schematic diagrams and scanning electron microscope (SEM) images of the electrodes. **a** Mushroom-shaped electrode. Reprinted and modified from Ref. ^49^ with permission from Frontiers; Ref. ^48^ with permission from Wiley. **b** Nanopillar electrode. Reprinted and modified from Ref. ^43^ with permission from Springer Nature; Ref. ^52^ with permission from American Chemical Society. **c** Nanowire electrode. Reprinted and modified from Ref. ^40^ with permission from Springer Nature. **d** iridium oxide (IrOx) nanotube electrodes. Inset: Top view showing the hollow center. Reprinted and modified from Ref. ^58^ with permission from Springer Nature; Ref. ^60^ with permission from American Chemical Society. **e** Volcano-shaped electrode. Reprinted and modified from Ref. ^42^ with permission from the American Chemical Society. **f** Nanocrown electrode. Inset: Enlarged view of the electrode tip. Reprinted and modified from Ref. ^64^ with permission from Springer Nature

Since the pioneering work by Thomas et al. in 1972, NEAs have garnered significant attention for the study of excitable cells, particularly cardiomyocytes^45–47^. 3D structures provide significant advantages by enhancing cell adhesion to the nanoelectrode surface, which in turn reduces the electrode’s exposed area and minimizes contact with the surrounding electrolyte solution during recordings. Figure 2 depicts various representative configurations of electrodes as well as the interface between 3D electrodes and cells. To improve coupling between external electrodes and nerve cells by mimicking the neuronal environment, Spira et al. developed gold mushroom-shaped microelectrodes (gMμEs), which typically comprise a stalk, a base, and a cap (Fig. 2a)^47–49^. The fabrication of gMμEs begins by coating silicon or glass samples with a Cr/Au layer using sputtering. Then, samples are spin-coated with photoresistant material. Photolithography, utilizing a photomask, can create openings in the photoresistor. gMμEs are subsequently formed on the surface through gold deposition, before removing the photoresistor layer. The quality of the pattern layer and the stalk thickness are determined by lithography parameters, while the mushroom head size depends on deposition conditions. gMμEs are effective for long-term detection of intracellular APs with improved endocytosis when coated with laminin, allowing for 2–10 min recordings under short electroporation pulses^47^. However, gMμEs have limitations. One issue is that the success of endocytosis can vary depending on where the cell is located. This variability affects the deformation of the membrane and the quality of the recordings. Additionally, high resistance at the junction between the membrane and electrode can lead to signal loss, which reduces the quality of detection. Solutions to these issues include fabricating a fence structure around the gMμEs to stabilize cell positioning and using low-impedance materials to reduce resistance and improve overall performance^48,49^. Recent research has demonstrated that vertically aligned nanopillar electrodes can establish strong interactions with cell membranes, significantly reducing impedance by several orders of magnitude through localized electroporation^44,50–53^. Nanopillar electrode fabrication involves photolithography for defining Pt tracks and contact pads on quartz substrates, followed by SiO_2_ deposition via chemical vapor deposition (CVD) under low-pressure conditions for insulation. Reactive ion etching and electron beam lithography create nanoholes above each Pt pad until the Pt layer. Next, Pt nanopillars can be deposited into the nanoholes using a focused ion beam (FIB). Finally, the devices are treated with oxygen plasma for cleaning before coating with a diluted Matrigel matrix and cell plating^43^. However, nanopillar electrode arrays can also be fabricated through a templated process including atomic layer deposition (ALD), etching, lithography, deposition, and liftoff. The electrodes feature a polyethylene terephthalate (PET) substrate with an Al_2_O_3_ core and a Ti/Au conductive coating, insulated with a negative photoresistor layer(Fig. 2b)^51^. Although nanopillar electrodes offer minimally invasive intracellular recording and are easily scalable, they can suffer from signal loss due to the gap between membrane and electrode. On the other hand, nanowires are ideal as intracellular probes due to their small size, which allows for minimally invasive insertion, while their solid-state nature prevents cell leakage^40,54–56^. Another process involves the fabrication of nanowire electrodes by plasma enhanced CVD (PECVD) for SiO_2_ layer deposition, stepper lithography, and dry etching to define SiO_2_ pillars, followed by wet etching to create the nanoelectrode core (Fig. 2c)^40^. Then, Ti and Pt are sputtered onto the pillars, before adding an ALD passivation layer. A resistor protects the base during wet etching, leaving the metal tip exposed for electrical interrogation. The latest vertical ultrasharp nanowires feature increased height, with tip diameters as small as 10 nm, enabling better cell penetration^57^. In other cases, iridium oxide (IrOx) nanotubes have been fabricated by cutting a quartz wafer into small pieces, followed by photolithography, Pt/Ti deposition, and liftoff to produce a pattern of electrode pads and lead lines^58^. The substrate is then coated with Si_3_N_4_ and SiO_2_ layers via PECVD. Plasma etching then exposes the Pt contact pads, and electron beam lithography defines nanoholes on a resist-coated chip. Then, the underlying Si_3_N_4_/SiO_2_ layers are removed to expose the Pt layer, and IrOx nanotubes are electrodeposited into the nanoholes using the resistor as a template (Fig. 2d). The advance of nanotube electrodes leverages cells’ natural propensity to interact with their environment^59^. In contrast to vertical nanopillar electrodes, the membrane protrusions within nanotube electrodes enhance local membrane tension and reduce the gap between the membrane and electrode^60,61^. Additionally, ion flows from ion channels or openings within the internal nanotube membrane are more readily detectable before dissipating into the surrounding medium. However, intracellular recordings using IrOx nanotube electrodes suffer from poor SNR and diminished signal amplitude, so addressing these issues is essential for achieving signal quality comparable to that of glass micropipettes and patch clamps^60^. Similarly, the fabrication of volcanic nanopatterned electrodes begins with successive evaporations of Ti, Pt, Ti, Au, and Ti layers onto a glass wafer, followed by SiO_2_ sputtering^42,62^. The substrate is then spin-coated with a photoresistor layer, patterned with 2-μm-diameter openings using a direct laser writer, and Ar^+^ ion beam etching, followed by photoresistor stripping with O_2_ plasma^42^. Electrically conductive tracks are then patterned on a new photoresistor layer, reflowed, placed at an angle, and stripped with O_2_ plasma. Finally, an insulating SU8 layer is spin-coated to complete electrode fabrication (Fig. 2e). Compared with other nanoelectrodes, nanovolcano (NV) electrodes, with a high aspect ratio and sharp design, offer improved cell access without reducing the effective recording area, leading to lower impedance and higher SNR. Recently, NV electrodes with a large 3D Pt electrode and an insulating SiO_2_ layer have been developed for stable intracellular recordings lasting >60 min^42^. Although functionalized gold nanoring was considered critical for sealing, later studies highlighted that tip geometry is more crucial for ensuring a secure cell-electrode interface^62^. Further research suggests optimizing the shape, size, and materials of NV electrodes, or integrating them with CMOS amplifiers, to enhance recording quality and duration^63^. Nanocrown electrodes (Fig. 2f) are partially hollow structures featuring an uneven crown edge^28,64^. The fabrication involves patterning Cr disks via photolithography and using reactive ion etching to form quartz nanostructures^64^. Ti and Pt are then deposited for metal connections, followed by Si_3_N_4_ and SiO_2_ insulation layers. Afterward, a wet etch reveals the nanoelectrode tips and further etching creates nanocrown structures, completing the fabrication. The nanocrown’s shape has been shown to promote cell membrane wrapping around its surface, improving cell adherence to its core^64^. This design allows for approximately 60 min of intracellular access to human pluripotent stem cells-derived cardiomyocytes (hPSC-CMs). Additionally, nanocrowns at a depth of 180 nm could record intracellular signals with twice the amplitude compared to those at a depth of 450 nm^64^.

### Innovations in other nanoelectrode designs

Recent advancements in nanoelectrode technology have focused on overcoming the inherent challenges related to the complexity and high costs of conventional manufacturing processes^65^. A notable innovation was the development of nanobranched electrode arrays, fabricated using a combination of hydrothermal growth and micromachining^66^. These electrodes are characterized by dense nanobranches with a high aspect ratio, where each electrode measures 20 × 20 μm^2^. The individual branches have a tip diameter of approximately 200 nm and a root diameter of around 100 nm. This unique configuration enhances the interface between electrode and cell, significantly improving intracellular signal recording. In experimental studies, these nanobranched electrodes successfully recorded intracellular signals from cardiomyocytes for up to 105 min, demonstrating effectiveness in long-term cell recordings^66^. This capability makes nanobranched electrodes highly valuable for extended electrophysiological studies.

Another significant advancement was the fabrication of nanotrap matrix electrodes from porous PET membranes using microfabrication lithography and magnetron sputtering^67^. These nanotrap electrodes incorporate traps of varying sizes and densities, strategically designed to promote a tight seal between the cell membrane and the electrode. This tight seal enables self-protruding electroporation, improving the quality and stability of intracellular recordings. This design can extend recording times to approximately 100 min, further demonstrating the efficacy of advanced electrode configurations^67^.

In addition to these advanced designs, supramolecular self-assembly electrodes have emerged as a promising way to simplify fabrication and reduce the associated costs^68^. Unlike traditional 3D micro/nanoelectrodes that require complex micro/nano fabrication or solvothermal synthesis, supramolecular self-assembly relies on small organic molecules that efficiently self-assemble into various 3D nanostructures, such as lamellated nanosheets, thin nanobelts, and rod-like structures^68^. These structures significantly enhance the cell-electrode interface, increasing quality and prolonging intracellular recordings compared to conventional electrodes. The simpler, cost-effective fabrication process makes these electrodes particularly suitable for large-scale electrophysiological studies. Furthermore, the exploration of additional geometrical designs, such as nanorods, offers potential pathways for further enhancing intracellular recording technologies, ultimately paving the way for more accessible and scalable bioelectronic devices^65,69,70^.

Advanced materials such as graphene, MoS_2_, and conductive polymers exhibit exceptional biocompatibility, mainly due to their low cytotoxicity, high surface area, and flexibility, which facilitate efficient interaction and integration with cells^71,72^. These materials promote cell adhesion and growth while minimizing inflammatory responses, ensuring the long-term stability and functionality of the NEAs. Additionally, surface functionalization with bioactive coatings can further enhance cellular interactions by creating a more favorable microenvironment for cell attachment^73^. This functionalization makes NEAs ideal for chronic applications in electrophysiology and disease modeling, where sustained compatibility with living cells is crucial.

## Strategies for intracellular access

To achieve high-fidelity intracellular signals, the nanodevice probe must penetrate the plasma membrane and establish strong coupling to the membrane. Effective intracellular access should enable high-quality, long-term signal recording while preserving cell viability^38,39^. Intracellular access methods are broadly categorized into electrode surface modification and physical perforation techniques^74^. Electrode surface modification, including the use of phospholipids or hydrophobic monolayers, enhances cell adhesion and promotes spontaneous membrane penetration^75^. In contrast, physical perforation techniques, such as optoporation and electroporation, provide a controlled and precise approach by opening transient pores in the cell membrane.

### Electrode surface modification

Spontaneous penetration, driven by chemical processes such as endocytosis and adhesion, occurs naturally with minimal impact on cell viability, allowing cells to securely and intimately adhere to the electrode surface^76,77^. During this process, the lipid bilayer of the cell membrane undergoes a structural rearrangement, forming a tight and robust seal at the penetration site essential for stable intracellular recordings over time. Surface modifications, such as the application of phospholipids or hydrophobic self-assembled monolayers can further optimize this interaction^78–80^. These modifications are specifically designed to enhance the formation of high-resistance membrane seals, thereby improving electrode-cell coupling and promoting more consistent, higher-quality intracellular recordings. Additionally, the precise engineering of these surface coatings improves control over the spontaneous penetration of the electrodes into cells, further reducing signal variability and enhancing the stability and duration of the recorded intracellular signals^42^.

**Fig. 3:**
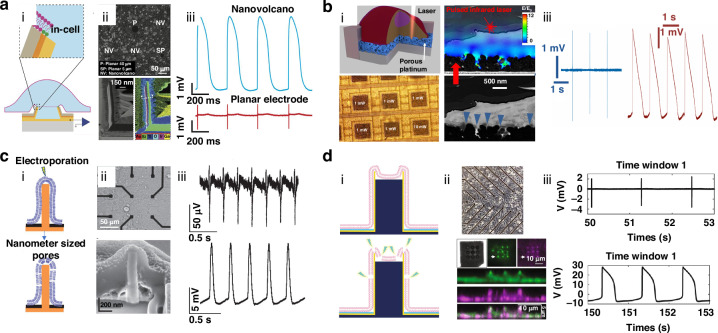
Representative penetration strategies for intracellular access. **a** (i) Chemical-induced spontaneous penetration indicated via a schematic of the Nanovolcano (NV)-cell interface, with dashed lines indicating the cell membrane at the interface where the cell contacts the electrode. (ii) Top, phase contrast image of a typical 3-day-old primary rat cardiomyocyte monolayer cultured on the NV array. Bottom, transmission electron microscopy (TEM) image alongside an energy-dispersive X-ray spectroscopy map of the redeposited multilayered wall. (iii) Extracellular and intracellular recordings. Reprinted and modified from Ref. ^42^ with permission from the American Chemical Society. **b** (i) Top, optoacoustic poration by planar meta-electrodes. Bottom, optical imaging of cells on a complementary metal oxide semiconductor (CMOS)-microelectrode array (MEA) after plasmonic optoacoustic poration. (ii) Bottom, SEM images showing cross-sections of HL-1 cells cultured and fixed on the meta-electrodes of a CMOS-MEA, blue arrows indicate the cell’s tightest adhesion points. Top, overlay with a model of the electric field distribution under laser exposure. (iii) Recordings of extracellular and intracellular signals before and after optoacoustic poration. Reprinted and modified from Ref. ^84^ with permission from Springer Nature. **c** (i) Electroporation facilitated by a nanopillar electrode. (ii) Top, brightfield images of HL-1 cells grown on a 3 × 3 grid of Pt pads. Bottom, interface between the cell and nanopillar electrode is uncovered through focused ion beam (FIB) milling, showing that the cell fully encapsulates the nanopillar electrode. (iii) Action potential (AP) recordings before and after electroporation. Reprinted and modified from Ref. ^44^ with permission from Springer Nature. **d** (i) Electroporation facilitated by nanocrown electrodes. (ii) Top, brightfield image showing a cardiomyocyte monolayer placed on a nanoelectrode array (NEA) device. Bottom, magnified view, enlarged view, and z-projection of a cell positioned on nanocrown electrodes, highlighting the membrane in green and integrins in purple across the vertical span of the nanocrown. (iii) AP recordings before and after electroporation. Reprinted and modified from Ref. ^64^ with permission from Springer Nature

For instance, hexanethiol-coated electrodes have shown remarkable potential by enabling spontaneous fusion with cell membranes, which supports long-term intracellular recordings (Fig. 3a)^42^. Although this spontaneous fusion significantly enhances recording duration, key challenges such as the complexity and labor-intensive nature of the modification procedures can make scaling up difficult^81,82^. Additionally, inefficiencies in consistently achieving reliable intracellular access further complicate the use of such electrodes. Spontaneous perforations, while beneficial, are often infrequent and short-lived, leading to difficulties in maintaining consistent and stable recordings^83^. These limitations highlight the need for further optimization of the electrode surface to improve both effectiveness and durability. Addressing these challenges is essential not only for enhancing the reliability of intracellular recordings but also for expanding their application potential to large-scale studies and broader research settings^38^.

### Physical perforation techniques

Physical perforation methods like optoporation offer significant advantages over chemically induced techniques, particularly in terms of precision and reduced cellular disruption^84,85^. Optoporation utilizes a focused laser pulse targeted at the electrode-cell interface, inducing various effects such as hot electron injection, thermal heating, and even bubble explosions, all of which facilitate the opening of transient pores in the cell membrane^86–88^. These methods, including plasmonic optoporation and optoacoustic poration, are designed to minimize disturbances to normal cellular activities, thereby supporting stable, long-term intracellular recordings. For instance, Dipalo et al. employed plasmonic optoporation together with vertical nanoelectrodes to generate transient nanopores, allowing uninterrupted intracellular recordings for up to 80 min (Fig. 3b)^84^. In another example, photoacoustic perforation by mechanical waves from optical pulses together with 3D fuzzy graphene electrodes allowed successful recording of intracellular electrical activity for 20 min^85^. Both techniques demonstrate a more controlled, less invasive approach to intracellular access, enhancing recording quality and duration without significantly compromising cell viability. As such, optoporation methods show great promise in the development of reliable, long-term intracellular recording platforms, contributing to advanced electrophysiological research and related applications^89^. However, achieving high-throughput parallel regulation using optoporation methods remains challenging. Integrating a 3D moving platform under the microscope provides a viable solution for enabling multisite optoporation, facilitating precise, high-throughput control of cells.

Electroporation, a widely used technique for intracellular recordings, involves applying electrical pulses to the cellular membrane, creating transient and localized pores that enable extracellular electrodes to capture APs^90^. Bio-membrane perforation in response to electrical stimuli was first documented in 1972; since then, electroporation has been a leading method in nanoelectrode research due to its simplicity, efficiency, and cost-effectiveness^45,91^. Electroporation is particularly valued for its ability to achieve intracellular access with minimal complexity. For instance, applying biphasic electrical pulses to HL-1 cardiac muscle cells cultured on nanopillar electrodes enables 10 min intracellular AP recordings (Fig. 3c)^44^. In a more refined approach, Jahed et al. developed a hollow-nanocrown electrode, which not only enhanced electroporation but also achieved a remarkable 99% success rate, significantly extending both the duration and amplitude of intracellular recordings^64^. Building on this progress, Fang et al. employed a scalable electroporation strategy using hollow nanopillar electrodes, extending intracellular AP recording durations to 100 min^70^. In addition, the concurrent recording of both intracellular and extracellular APs using 3D NEAs via electroporation has gained considerable attention. For example, Hu et al. successfully recorded intracellular and extracellular signals from neonatal rat cardiomyocytes using 3D nanobranched electrodes^66^. While electroporation substantially enhances the amplitude and quality of intracellular signals, it has some limitations. The process may temporarily disrupt the cell’s electrophysiological functions and the duration of intracellular access is constrained by the cell membrane’s natural repair mechanisms, which try to quickly reseal the transient pores created during the procedure^62,92,93^. Despite these challenges, electroporation remains a powerful tool in electrophysiological research, continuously contributing to the development of more effective recording technologies.

## Applications of 3D nanodevices for cardiac electrophysiology

Arrhythmias are closely associated with AP function in cardiomyocytes, having a key role in maintaining the heart’s rhythmic contractions^94,95^. Proper AP generation and propagation depend on the precise regulation of cardiac ion channels; any disruption in this regulation can result in abnormal heart rhythm or arrhythmia^96^. Thus, a deep understanding of cardiomyocyte APs is essential for both pharmacological testing and disease modeling, as it allows researchers to study how different drugs or genetic conditions might affect cardiac function. Traditional extracellular recordings, while useful, tend to produce low-resolution signals that fail to capture the full complexity of the waveform, often lacking key information on ion channel dynamics^69,97^. In contrast, intracellular AP recordings from 3D electrodes offer much more detailed data, providing critical insights into the resting membrane potential and specific ion channel activity^98–100^. Thus, passive 3D nanoelectrodes are particularly valuable in advanced cardiology research, enabling higher-resolution recordings crucial for accurately assessing cardiomyocyte function and investigating the underlying mechanisms of arrhythmias (Table 2).

**Table 2:** Summary of passive 3D nanoelectrode applications for cardiac electrophysiology

Geometry	Fabrication method	Materials	Throughput	Access strategies	Maximum AP (mV)	Duration	Cells	Application	Refs.
gMμEs	CVD, evaporation, photolithography, gold electroplating, etching	Ti-Au- SiO_2_	62	Electroporation	6	2–10 min	Rat primary cardiomyocytes	-	^48^
Vertical nanopillar	PECVD, FIB, photolithography	Pt-Ti-Si_3_N_4_-SiO_2_- Pt	16	Electroporation	11.8	10 min	HL-1 cells	Drug screening	^44^
Nanopillar	CVD, EBL, FIB, etching	Pt-SiO_2_	-	Electroporation	25.15	-	hPSC-CMs	Drug screening, Modeling disease	^43^
Nanopillar	FIB, deposition, etching	Au-SU8-Pt-SiO_2_	16	Electroporation	2.3	~10 min	HL-1 cells	Modeling disease	^118^
Nanopillar	ALD, photolithography, etching	PET-Al_2_O_3_-Au-Ti-SU8	-	Electroporation	0.2216 ± 0.2175	-	Neonatal rat cardiomyocytes	Drug screening	^54^
IrOx nanotube	PECVD, EBL, photolithography, etching, deposition	Pt/Ti-Si_3_N_4_-SiO_2_-IrOx	60	Electroporation	15	~60 min	HL-1 cells, primary rat cardiomyocytes	-	^60^
Vertical NW	PECVD, ALD, photolithography, etching	SiO_2_-Ti-Pt-SiO_2_	1024	Electroporation	20	-	Rat ventricular cardiomyocytes	Drug screening	^40^
Ultrasharp NW	PECVD, EBL, photolithography, etching	Si-SiO_2_-Pt-SiO_2_	4	Spontaneous penetration	60	-	iPSC-derived cardiovascular progenitor cells	-	^59^
3D plasmonic nanoelectrode	FIB, evaporation	Ti/Au-SU8-SiO_2_-Polyimide	-	Plasmonic optoporation	1.5	~80 min	HL-1 cells	-	^52^
Nanovolcano	EBL, photolithography, deposition, etching	Ti-Pt-Ti-Au-Ti-SiO_2_-SU8	32	Spontaneous penetration	20	Over 1 h	Primary rat cardiomyocytes	-	^42^
Nanobranched	Photolithography, hydrothermal growth	ZnO-Cr/Au or Pt-SU8	16/32	Electroporation	~5	105 min	Rat cardiomyocytes	-	^69^
Nanorods	Photolithography, hydrothermal growth	ZnO-Cr-Au(or Pt)-SU8	32	Electroporation	~5	~80 s	Neonatal rat cardiomyocytes	-	^72^
Nanocrown	FIB, deposition, etching	Cr-Ti-Pt-Si_3_N_4_-SiO_2_	58	Electroporation	~63	~60 min	hPSC-CMs	Drug screening	^66^
Nanotrap	Photolithography, deposition	PET-Ti-Au-SU8	32	Electroporation	4.32	~100 min	Neonatal rat cardiomyocytes	Drug screening	^70^
Nanowell	FIB, photolithography, deposition, etching	Si-Cr-Au-SiO_2_-Ni	16	Electroporation	~0.1	10–20 min	HL-1 cells	-	^68^
Vertical nanotemplate	ALD, photolithography, etching	PET-Al_2_O_3_-Ti-Au-SU8	32	Electroporation	6.97	10 min	Primary neonatal rat cardiomyocytes	Drug screening	^53^
Hollow nanopillar	ALD, photolithography, etching	PET-Al_2_O_3_-Ti-Au-SU8	32	Electroporation	1.5	Over 100 min	Primary rat cardiomyocytes	Drug screening	^73^
Supramolecular self-assemblies	Evaporation, deposition	Ti-Au-SiO_2_- SS-1, or SS-2, or SS-3	16	Electroporation	~4.0	10 min	Primary neonatal rat cardiomyocytes	-	^71^

*gMμEs* gold mushroom-shaped microelectrodes, *CVD* chemical vapor deposition, *PECVD* plasma enhanced CVD, *FIB* focused ion beam, *EBL* electron beam lithography, *hPSC-CMs* human pluripotent stem cells-derived cardiomyocytes, *ALD* atomic layer deposition, *IrOx* iridium oxide, *NW* nanowire

### Cardiomyocyte-based passive 3D nanodevices for drug screening

**Fig. 4:**
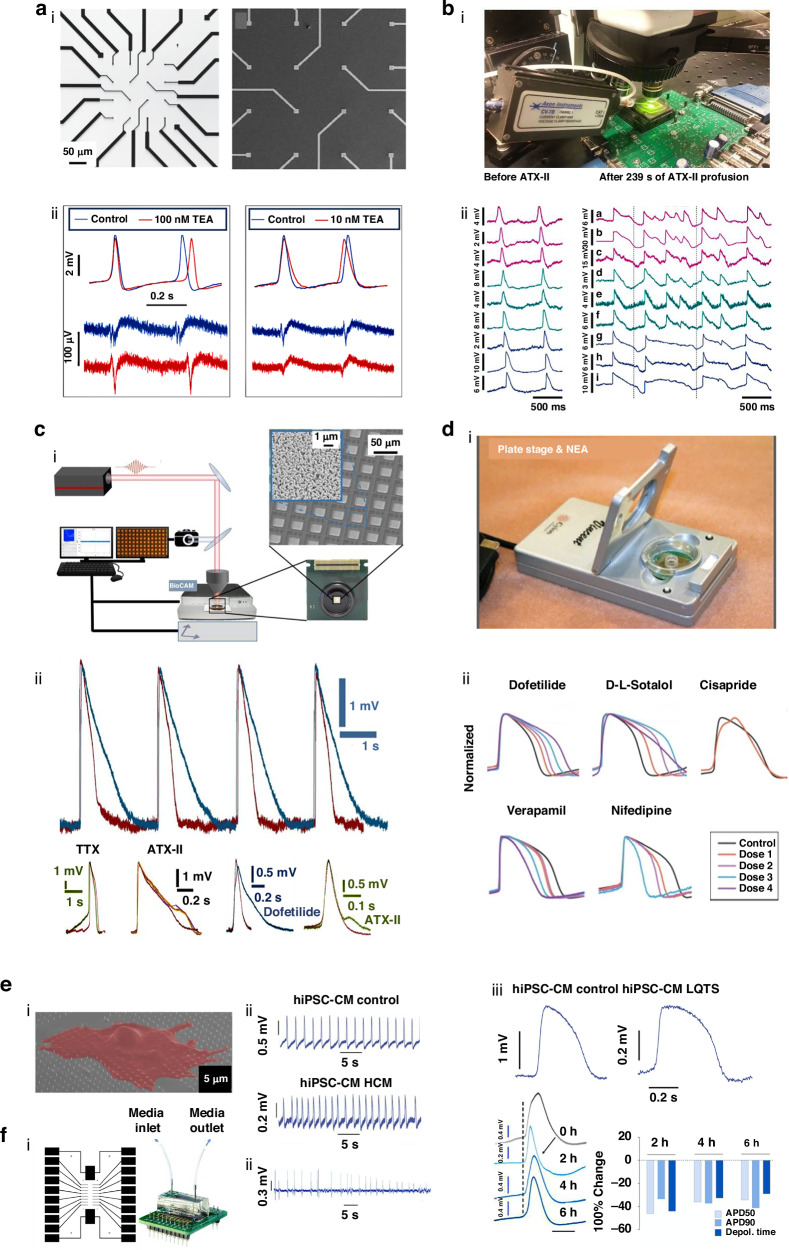
3D bioelectronic platforms for drug testing and disease modeling. **a**–**d** Applications of 3D NEAs for drug screening. **a** Intracellular APs recorded by nanopillar electrodes after administration of nifedipine and TEA. Reprinted and modified from Ref. ^44^ with permission from Springer Nature. **b** Intracellular APs recorded using CMOS-NEA before and after ATX-II administration. Reprinted and modified from Ref. ^40^ with permission from Springer Nature. **c** Large-scale optoacoustic poration and intracellular recording in cell networks. Reprinted and modified from Ref. ^84^ with permission from Springer Nature. **d** Nanocrown NEA device and intracellular recordings before and after drug administration at varying concentrations. Reprinted and modified from Ref. ^64^ with permission from Springer Nature. **e** Intracellular recordings from hiPSC-CMs using nanopillars, showcasing data from a healthy individual and a patient with hypertrophic cardiomyopathy and long QT syndrome. Reprinted and modified from Ref. ^43^ with permission from Springer Nature. **f** Intracellular recordings displaying arrhythmic activity following prolonged hypoxic stress. Reprinted and modified from Ref. ^114^ with permission from the American Chemical Society

Recent advances in drug development have increased the prevalence of cardiotoxicity-related issues, leading to drug withdrawal and failures in clinical trials^101,102^. Certain classes of drugs, particularly anticancer treatments, and antibiotics such as propafenone, flecainide, and terbutaline, have been closely associated with severe arrhythmias due to their significant impacts on cardiac ion channels^103^. Drug-induced cardiotoxicity can be acute and/or chronic, posing considerable challenges to long-term patient safety and requiring more reliable and efficient cardiotoxicity evaluation methods^102,104^. As a result, interest in the use of NEA platforms for in vitro drug screening has risen, as these platforms enable the detection of subtle changes in cardiomyocyte APs and ion channel activity in response to drug exposure (Fig. 4)^18,98,105–107^.

Nanodevices employing passive 3D NEAs are emerging as powerful tools in drug screening, providing critical insights into how drugs impact cardiac ion channels and APs^108^. For instance, Xie et al. utilized nanopillar electrode arrays to investigate the effects of nifedipine, a calcium channel blocker, and tetraethylammonium (TEA), a potassium channel blocker, on APs in HL-1 cells^44^. A concentration of 100 nM nifedipine caused a significant reduction in APD50 and extended the refractory period, whereas 10 mM TEA had the opposite effect (Fig. 4a). Further, Abbott et al. developed a CMOS-NEA biosensing platform with 1024 channels allowing for extensive and high-resolution intracellular recordings^40^. After perfusing cells with ATX-II for 239 s, they observed a uniform increase in AP duration across a cellular sheet, with notable regional differences in the extent of APD prolongation (Fig. 4b). Building on this finding, Dipalo et al. integrated CMOS-NEA technology with planar porous electrodes to enhance functionality^84^. Human-induced pluripotent stem cells were treated with 500 nM dofetilide, 50 μM tetrodotoxin (TTX), and 50 nM ATX-II, which revealed distinct cellular responses, such as slower repolarization (dofetilide), prolonged depolarization (TTX), and altered cellular shapes (ATX-II; Fig. 4c). Moreover, nanocrown electrode arrays demonstrated exceptional sensitivity to subtle drug-induced changes in cardiac electrophysiology, due to fast-sampling rates and self-referential comparison capabilities^64^. Jahed et al. developed a 57-electrode nanocrown array, which successfully differentiated drug responses, showing that high-risk drugs like dofetilide (0.3 nM, 1 nM, 3 nM, 10 nM) and D,L-Sotalol (0.1 µM, 1 µM, 10 µM, 100 µM) cause a dose-dependent increase in AP duration, while lower-risk drugs such as verapamil (1 nM, 10 nM, 100 nM, 1000 nM) and nifedipine (1 nM, 10 nM, 100 nM, 1000 nM) decreased AP duration (Fig. 4d)^64^. These advances highlight the potential of passive 3D NEAs in providing detailed and reliable data for drug screening and cardiac safety assessment.

### Passive 3D nanodevices for advanced cardiac disease modeling

In vitro modeling has emerged as an essential tool for creating precisely controlled environments that replicate organ and tissue structures with high accuracy^109^. Advances in cell culture technologies, including cardiomyocyte cultures, stem cell technologies, and 3D tissue engineering, have enabled the cultivation of myocardial tissue that demonstrates synchronized beating, closely mimicking in vivo behaviors^110–112^. When integrated with micro-nano fabrication techniques, biomedical technologies provide researchers with powerful platforms for large-scale investigation of disease mechanisms, offering critical insights to drive significant advancements in the understanding of CVDs^113^.

Passive 3D NEAs show significant potential for advancing disease modeling, offering more precise and detailed studies of cellular behaviors under pathological conditions. Lin et al. developed an innovative disease modeling platform using a 3 × 3 array of vertical Pt nanopillars to study hPSC-CMs^43^. The system enabled the simultaneous recording of intracellular APs using nanopillar electrodes together with a conventional patch clamp, providing a more comprehensive view of the cells’ electrical activity. The platform was particularly effective in modeling hypertrophic cardiomyopathy, as it accurately captured arrhythmic activity and delayed depolarization patterns, in contrast to the regular beat intervals observed in healthy cardiomyocytes^43^. Moreover, the platform successfully demonstrated the prolonged APs characteristic of long QT syndrome, further validating its utility in disease-specific research^43^. In another study, Liu et al. designed a heart-on-a-chip model to investigate the effects of acute hypoxia on cardiac performance^114^. This advanced model incorporated a microfluidic channel, a cell culture zone, and embedded electronic components capable of recording both extracellular and intracellular electrical signals. Under hypoxic conditions (1% O_2_) over a 6-h period, the platform revealed significant decreases in APD50, APD90, and depolarization time in cardiomyocytes, demonstrating the system’s sensitivity to changes in the cellular environment. These effects persisted throughout the study (at 0, 2, 4, and 6 h), providing valuable insights into how hypoxia impacts cardiac electrical activity over time^114^. Future advances in passive 3D NEAs are expected to involve the integration of CMOS circuits, enabling the development of high-throughput, high-resolution electrophysiological platforms to simultaneously study multiple parameters, offering a more detailed investigation of cellular activities^115,116^. Additionally, the combination of passive 3D NEAs with tissue engineering technologies holds the potential to create more accurate and biomimetic 3D tissue models for disease studies^117,118^. Such systems, equipped with multiparameter sensing and regulatory capabilities, could pave the way for comprehensive investigations into the physiological mechanisms that govern cellular functions, ultimately contributing to better understanding and treatment of complex cardiac diseases^25,119^.

### Perspective and conclusion

Emerging trends in micro-nano-bioelectronics, with 3D NEAs at the forefront, are shaping the future of intracellular recordings, particularly in cardiac research. These advanced devices hold tremendous potential for more precise and high-resolution insights into cardiac activity, helping to uncover new therapeutic approaches for heart diseases. However, several challenges must be addressed to fully realize the promise of passive 3D NEAs. First, further optimizing the design and penetration techniques of passive NEAs is critical for achieving efficient interfacing with cardiomyocytes^35,108,120^. Electroporation faces challenges such as membrane disruption, inconsistent permeabilization, and noise interference during intracellular recordings^41^. Single-pulse electroporation is considered a less invasive method for the study of cardiomyocytes; however, precise regulation of parameters such as pulse amplitude and duration is critical for enhancing recording fidelity^64,121^. Advancing single-pulse techniques and integrating advanced noise-reduction strategies are vital for achieving stable, safe, and high-quality intracellular recordings. Second, integration of NEAs with CMOS technology, which has the potential to enable high-throughput, high-resolution intracellular recordings at a large scale^122–124^. CMOS integration significantly improves spatial resolution by minimizing the number of leads at the electrode layer, achieved through shared leads, allowing for thousands of electrodes to be integrated. Leveraging addressable capabilities, these passive NEAs enable recordings at both the single cells and their networks, allowing for precise cell modulation and the detailed mapping of electrical activity propagation within the cells^40^. Furthermore, combining passive NEAs with CMOS technology boosts scalability by enabling high-density electrode integration, parallel data processing, miniaturization, and cost-effective production, making the system suitable for large-scale applications^125^. However, while CMOS integration enhances functionality, new technical challenges arise, such as the need for innovative solutions to maintain signal fidelity, manage heat dissipation, and ensure seamless integration of the two technologies^123,125,126^. Consequently, material selection and structural precision remain critical factors^127–129^. Ongoing research focuses on refining manufacturing methods and material properties to improve the overall performance of these devices^38^. Compared to traditional inorganic electrodes, new soft and functionalized materials offer significant potential for enhancing the biocompatibility of NEAs. To effectively incorporate these promising biocompatible materials into passive 3D NEAs, developing optimized fabrication techniques that suit their unique properties is essential. Conventional fabrication methods often lack compatibility with soft materials, necessitating the development of innovative dry patterning, low-temperature deposition, and plasma-free processes that are compatible with novel materials^130^. Certain 2D materials are prone to oxidation in biochemical environments, necessitating the development of surface passivation techniques that not only maintain electrical conductivity but also provide effective insulation against water and oxygen. These advancements would not only improve biocompatibility but also enhance the long-term stability of the devices. Furthermore, future developments in multiparameter detection aim to revolutionize the use of 3D nanoelectrodes, particularly for drug toxicity assessments^69,100,131^. By enabling simultaneous monitoring of multiple biological parameters, the reliability and effectiveness of the drug screening platforms could be significantly enhanced^99,132–134^. The use of organoids will further strengthen these drug testing systems, providing a more accurate representation of how drugs interact with human tissues^135–138^. Beyond drug screening, integrating bioelectronic devices with electrical stimulation technologies offers promising new treatments for cardiac conditions such as arrhythmias^139^. By applying targeted electrical impulses to heart tissues, these devices could help restore normal heart rhythms^140^.

In summary, passive 3D NEAs represent promising tools for cardiovascular research and drug development, offering precise, long-term intracellular recordings of cardiomyocytes. Integration with microfluidic systems, CMOS technology, and real-time drug delivery will transform the landscape of CVD research and treatment. In the future, advances in materials, microfluidics, and chip technologies will drive the development of multifunctional, integrated diagnostic and therapeutic platforms for CVDs.
